# Body composition from birth to 2 years

**DOI:** 10.1038/s41430-023-01322-7

**Published:** 2023-08-10

**Authors:** Andrew P. Hills, Shane A. Norris, Nuala M. Byrne, Sisitha Jayasinghe, Alexia J. Murphy-Alford, Cornelia U. Loechl, Leila I. Cheikh Ismail, Anura V. Kurpad, Rebecca Kuriyan, Lukhanyo H. Nyati, Ina S. Santos, Caroline S. Costa, V. Pujitha Wickramasinghe, M. Nishani Lucas, Christine Slater, Ayesha Yameen, Shabina Ariff, Multi-center Infant Body Composition Reference Study (MIBCRS)

**Affiliations:** 1 University of Tasmania, Launceston, Australia; 2 University of the Witwatersrand, Johannesburg, South Africa; 3 University of Southampton, Southampton, United Kingdom; 4 International Atomic Energy Agency, Vienna, Austria; 5 University of Sharjah, Sharjah, United Arab Emirates; 6 University of Oxford, Oxford, United Kingdom; 7 St. John’s Research Institute, Bangalore, India; 8 Federal University of Pelotas, Pelotas, Brazil; 9 University of Colombo, Colombo, Sri Lanka; 10 Pakistan Institute of Nuclear Science and Technology (PINSTECH), Nilore, Pakistan; 11 Aga Khan University, Karachi, Pakistan

**Keywords:** Translational research, Obesity

## Abstract

Providing all infants with the best start to life is a universal but challenging goal for the global community. Historically, the size and shape of infants, quantified by anthropometry and commencing with birthweight, has been the common yardstick for physical growth and development. Anthropometry has long been considered a proxy for nutritional status during infancy when, under ideal circumstances, changes in size and shape are most rapid. Developed from data collected in the Multicentre Growth Reference Study (MGRS), WHO Child Growth Standards for healthy infants and children have been widely accepted and progressively adopted. In contrast, and somewhat surprisingly, much less is understood about the ‘quality’ of growth as reflected by body composition during infancy. Recent advances in body composition assessment, including the more widespread use of air displacement plethysmography (ADP) across the first months of life, have contributed to a progressive increase in our knowledge and understanding of growth and development. Along with stable isotope approaches, most commonly the deuterium dilution (DD) technique, the criterion measure of total body water (TBW), our ability to quantify lean and fat tissue using a two-compartment model, has been greatly enhanced. However, until now, global reference charts for the body composition of healthy infants have been lacking. This paper details some of the historical challenges associated with the assessment of body composition across the first two years of life, and references the logical next steps in growth assessments, including reference charts.

## Introduction

Historically, the growth, development and nutritional status of infants has been based on anthropometric measurements, most commonly, length (height), weight (mass) and head circumference [1–3]. Infancy has long been recognized as one of the most vulnerable life stages but more recently, the early years have been identified as critical in framing phenotypic metabolic profiles associated with health status in later life [4–9]. A focused lens on the early years also includes widespread acknowledgement of the importance of a healthy pregnancy and lactation for optimal fetal and infant growth, respectively [10]. Associations between birth size and susceptibility to ill-health as an adult suggest that the growth and development of the infant, including ‘quality’ of growth, is integral to the risk of later abnormal metabolic function. A better understanding of early life changes is particularly important given the dire forecasts regarding the increasing prevalence of cardiovascular diseases, numerous cancers, type 2 diabetes, and bone disease in coming decades. In each case, these disorders are associated with overweight and obesity [8, 11].

## The first 1000 days – a critical window

The first 1000 days, from conception to 2 y of age, is widely referenced as a ‘critical window’ for growth and development. During this period, a significant part of the trajectory of an individual’s health status is shaped, and it may also represent the most opportune time for intervention [6, 12]. The ability to assess both growth and body composition during this period may greatly assist in the early identification of health risk, and with appropriate intervention, impact the potential of progression to ill-health [13, 14]. As a foundation, the aggregation of normative body composition and anthropometric data on infants, and ideally, during fetal growth, would enable the further characterization of a healthy pregnancy and quantify the quality of growth. Logic suggests that healthy growth and development is not limited to the first 1000 days but also includes pre-conception. This adds weight to the fact that the growth, body composition and health status of adolescents and young women, ahead of the childbearing years, also deserves closer attention [6, 15, 16].

## The current state of play

Despite recognition of the fundamental importance of a good start to life, malnourishment and undernutrition are still commonplace in many mothers and young children [17–20]. Maternal and child undernutrition is estimated to account for approximately 3·5 million deaths (~11% of global DALYs) in children under 5 y, with stunting, severe wasting, and intrauterine growth restriction constituting the main risk factors [17, 21, 22]. Sadly, these trends are disproportionately higher in low- to middle-income countries, largely due to lifestyle and dietary factors [22–24]. In many settings, including in Africa and South Asia, the ‘double burden of malnutrition’, the combination of suboptimal nutrition and overweight, or the ‘triple burden’, when micronutrient deficiencies also exist, are widespread [20, 23, 25–32].

In short, a poor start to life can have irreversible effects, including shorter adult stature, lower school achievement, reduced adult income, and lower birth weight of offspring [20, 22, 33]. Individuals who are small at birth and remain short but become obese during childhood, have an increased risk of co-morbidities of overweight and obesity [34–36]. The epidemiological evidence associating smaller size or relative thinness at birth and during infancy with higher rates of chronic disease in adulthood [37–39], is particularly strong [6, 40]. For example, increased risk of type 2 diabetes, hypertension, and obesity is greater in individuals with low birth weight and thinness at 2 y of age followed by rapid weight gain [4, 41–44], and under-nourishment early and overweight later in life, is commonly associated with earlier onset and more severe symptoms than individuals who were never undernourished. Sachdev and colleagues [45] recently cited the very concerning intraindividual double burden of undernutrition (as defined by anthropometry), and ‘metabolic obesity’ in a large study of Indian children. In normal and undernourished groups in this study, a high proportion of children had elevated cardiometabolic risk markers.

A major shortcoming of many studies has been a lack of objective body composition data and an overreliance on body weight and length [46] and indices such as BMI [40]. A number of studies [14, 47] have referenced the use of height, weight and skinfold thicknesses as surrogate methods to ‘assess’ body composition. Without quantification of body composition, we cannot be confident that changes in anthropometry, including BMI, are related to differential adiposity [8, 48]. Limitations of BMI have also been cited in relation to individuals of different ethnicity [6], and resulted in some inconsistences, for example, the ‘thin-fat’ phenotype reported in South Asian infants in some studies [49], but not others [13]. In summary, differences in relative adiposity across ethnic groups has been largely based on anthropometric measures and we are yet to fully understand the nutritional and wider environmental factors influencing such differences.

Greater clarity regarding the interrelationships between diet, nutrition, physical activity, body composition, functional capacity during infancy and childhood, and risk of later disease, is essential, particularly considering regional nuances [50, 51]. The size and shape, plus body composition of an individual, reflects the extent to which the amount and quality of the food consumed over an extended period has adequately (or not) met the needs of the body. As Lopez et al. [52] indicated, it is not surprising that poor nutrition is a major risk factor for disease. Indeed, it is regrettable that *‘nutrition is* (still) *a desperately neglected aspect of maternal, new-born and child health’* [53], along with quantification and monitoring of body composition [8].

## Limitations in the traditional assessment of physical growth and development

A wide range of approaches have been used to assess the size, shape and/or composition of the body and its component parts. Anthropometric screening of height and weight, and the relationship between these measures at different ages, has also been used as a crude indicator of body composition, expressed as BMI, ponderal index or weight for height. Despite the extensive use of body weight as a marker of growth, commencing with birthweight, the relative proportions of lean and adipose tissue can be highly variable for the same body weight [6, 54, 55]. This implies that even at the simplest level, our understanding of the factors that determine the relative partitioning of nutrients in a 2-compartment model (fat mass [FM] and fat-free mass [FFM]), is limited.

At a population level, anthropometry is a convenient approach to monitor changes in physical growth and may also provide an approximation of corresponding changes in body composition. However, one of the factors that compromises the utility of anthropometric measures, including existing data on pregnant women and infants, relates to the lack of representativeness to all population groups [56, 57]. Similarly, the accuracy and precision of measurements should be optimal if anthropometry is utilized to assess relationships between early nutrition and longer-term health. There is also a lack of acknowledgement by some that anthropometric measures and indices are not measures of body composition per se, despite being convenient correlates of FM, FFM and bone mass, key indicators of adequacy of infant nutrition [58]. Noting the limitations, standardized anthropometric approaches to quantify changes in size and shape of mothers and infants should not be underestimated. However, it would be much more valuable to overlay traditional size and shape measures with body composition data to better define the ‘quality’ of physical growth.

The human body can be divided into different components and at all ages, water represents the largest proportion. Under healthy conditions, most body water exists within the lean tissue and accordingly, an assessment of TBW provides us with an index of FFM. Assuming a 2-compartment model, FM can be derived by subtracting FFM from body mass. The simple but versatile two-compartment model has been widely used in nutrition science to estimate both FFM and FM. The model continues to have currency because despite the availability of more sophisticated technology, the use of 3- and 4-compartment approaches require additional technical equipment and therefore tend to be restricted to smaller sample sizes [25, 26, 59].

It is also worth highlighting that to date, most body composition assessment techniques have been developed for, and validated in, adults, meaning their direct application to infants and young children is questionable. Once mature adult status has been attained, under healthy conditions, body size and composition remain relatively constant over time. In contrast, the dynamic and complex nature of the growth changes during infancy are such that the standardization of measurement is particularly challenging. The field of body composition assessment has rapidly evolved in the past few decades and has resulted in the contemporary availability of a range of sophisticated techniques [25, 47, 55, 60–65]. Amongst a myriad of options, hydrometry, based on isotope dilution [66], is a particularly useful tool for compositional assessment in infancy. Being non-radioactive, safe, and relative ease of administration, are all factors that have contributed to the increased utilization of stable techniques in recent times [67].

Despite advances in body composition assessment techniques, detailed knowledge of the specific compositional changes during pregnancy and across the first two years, remains limited [62, 68]. A more comprehensive understanding of the normal variability in the body composition of the mother, neonate and infant has the potential to significantly improve maternal and infant health and be a major step forward for the global health agenda. Valid and reliable body composition data would significantly advance our knowledge and understanding of normal growth plus the impact of reduced height velocity in undernourished infants, along with greater detail regarding stunting [69, 70].

## Greater clarity regarding growth is not without its challenges

Assessment of growth in early life has many unique challenges [62], particularly given the assortment of intra-uterine and neonatal factors modulating fetal growth and tissue accretion at birth and into the early post-partum period [54, 55, 60]. Rapid fetal body composition changes late in a normal pregnancy include gains in both lean and adipose tissue, and weight [55, 60, 64].

Birth is characterized by fundamental changes in physiology and metabolism as the new-born adapts to an independent extra-uterine environment high in oxygen. Marked changes in weight and composition in the first 72 h of life [60, 71] include changes in the extracellular space, TBW and water levels in tissue fractions [72, 73]. For example, hydration of the FFM can decrease by about 5% across the first year of life and relative fat mass (%FM) changes from approximately 11–15% at birth to 30% at 6 months [72, 74–76]. This multiplies the potential complications in the accurate monitoring of growth and body composition during infancy.

As mentioned earlier, the prevention of chronic disease in later life, including co-morbidities of obesity, would greatly benefit from the ability to quantify body composition and track change over time. A better understanding of the specific contribution of intrauterine and post-natal periods to increased adiposity [34, 77–79] would be a significant advance. Another major advantage of detailed assessments of body composition during the early years would be to quantify the impact of interventions in this age group [19, 80, 81]. Likewise, it is important to focus more attention on premature infants whose growth is frequently stunted during the early postnatal period [63]. Although discharge weights and term-corrected statistics from around the world indicate that preterm babies typically make up for lost growth and catch up to their full-term counterparts, there is insufficient understanding of how FM and FFM contribute to this atypical growth pattern. Given the link between infant body composition in early life and cardiovascular, metabolic, and neurological outcomes, comprehending this process is crucial [82, 83].

A major evidence gap in the field is the lack of a comprehensive understanding of variability in growth between populations. Primarily, this shortcoming stems from the traditional bias for data collection in high-income countries [48]. Under normal, healthy or ‘ideal conditions’, infancy is a period of rapid linear growth with proportionate increases in FM and FFM [84].

## The value of high-quality, normative body composition data

Despite some ambiguity regarding definition, normative data commonly references an observed, preferred, statistical statement of distribution of characteristics in a defined population at a specific point in time. Traditionally, growth assessments have been made on representative convenience samples and less commonly on populations in which preferred health has been characterized. More recent efforts, for example the INTERGROWTH-21^st^ Consortium developed to ascertain if fetal growth, under optimal circumstances, has sufficient cross-population similarities to justify an international fetal and preterm postnatal growth standard, affirmed the appropriateness of such approaches [85]. The WHO Child Growth Standards developed from the MGRS are another excellent example in this context. These studies have made a significant contribution to our understanding of how children free of disease, should grow when reared following standard health practices including breastfeeding and infant and young child feeding (IYCF) practices in a non-smoking environment [86, 87]. Never before has the global community had the luxury of building on this foundation material with a complementary set of body composition data. The derivation of body composition reference charts for infants in the first 2 y of life represents the logical next step. The availability of the first normative body composition data, encompassing variability in the quality of growth of infants up to 2 y of age across different settings and ethnic groups, provides greater clarity regarding growth and development during this critical life stage [86].

## Conclusion

Recently published reference charts for body composition or ‘quality’ of growth are a welcome addition to WHO Child Growth Standards generated from anthropometric data. A logical next step is to develop global consensus on definitions and protocols for both anthropometry and body composition assessment in infancy. Such a development would be a major advantage for the world’s youngest and most vulnerable population. Harmonization and standardization of measurement approaches could be achieved through a shared vision for global partnerships between UN agencies and stakeholder organizations, and a systematic increase in quality control mechanisms, including the strategic education and training of relevant health professionals.

## Supplementary information

MIBCRS Consortia
