# Supplementary material for: Body composition from birth to 2 years
Source: Eur J Clin Nutr. 2023 Aug 10;78(11):923–7. doi: 10.1038/s41430-023-01322-7 (PMC11537967; doi:10.1038/s41430-023-01322-7)
Supplement: Supplementary file 1 — MIBCRS Consortia [file 41430_2023_1322_MOESM1_ESM.docx]

SUPPLEMENTARY MATERIAL

*Multi-center Infant Body Composition Reference Study (MIBCRS): Shabina Ariff, Andrew P Hills, Rebecca Kuriyan, Shane A Norris, Ina S Santos, V Pujitha Wickramasinghe, Alexia J Murphy-Alford, Lukhanyo H Nyati, Caroline S Costa, M Nishani Lucas, Tanvir Ahmad, Kiran DK Ahuja, Jeff M Beckett, Renata M Bielemann, Nuala M Byrne, Laila Charania, Michele P Christian, Divya J Priscilla, Anne Hanley, Manoja P Herath, Leila I Cheikh Ismail, Sisitha Jayasinghe, Anura V Kurpad, Pulani Lanerolle, Cornelia U Loechl, Najat Mokhtar, Upul Senarath, Christine Slater, Sajid Soofi, Steven J Street, Neiva CJ Valle, and Ayesha Yameen
